# Plastics Biodegradation in the Short Term in a Mediterranean Soil and the Effect of Organic Amendment

**DOI:** 10.3390/toxics13060486

**Published:** 2025-06-09

**Authors:** Rafael Boluda, Nadia Redondo, Luis Roca-Pérez, Eva Fernández-Gómez, Oscar Andreu-Sánchez

**Affiliations:** Departament de Biologia Vegetal, Facultat de Farmàcia, Universitat de València, Burjassot, E-46100 València, Spain; boluda@uv.es (R.B.); eva.fernandez-gomez@uv.es (E.F.-G.); oscar.andreu@uv.es (O.A.-S.)

**Keywords:** soil, plastic, biodegradation, manure

## Abstract

The main problem with the conventional plastics presently used is that they are too slow to degrade, and thus, accumulate in the natural environment. This situation occurs on farmlands because low-density polyethylene (LDPE) is widely used in agriculture. Different authors propose employing biodegradable plastics (bioplastics) to solve this problem, and the most studied and promising candidates are poly(hydroxybutyrate) acid (PHB) and poly(lactic) acid (PLA). This work centers on the short-term evaluation of the biodegradability of the three above-mentioned plastic materials in soil type Mediterranean Alfisol and the effect of adding organic amendment (cow manure; CM) on their biodegradation. Two experiments were run for each plastic material: one without this organic amendment and the other by adding CM. Their biodegradation was determined by the procedure described in Standard ISO 17556. The results confirm that PHB is a highly biodegradable polymer, whereas the biodegradability of PLA and LDPE is poor. Using CM did not facilitate plastic polymer biodegradation in our soil. The nature and properties of soil can significantly impact plastics biodegradation. Bioplastics are still not the panacea to solve the plastics pollution problem, so other management options must be considered, such as prevention, reduction, and/or reuse in situ.

## 1. Introduction

The presence of plastics in the natural environment was detected for the first time at the beginning of the 20th century [1]. Since then, plastics production has considerably grown by rising from 2 million tons in 1950 to 400.3 million tons in 2022 [2]. Plastics were originally synthetic organic polymers that were derived from the polymerization of monomers extracted from fossil fuels (oil or gas) [3]. Nowadays, plastics can be classified into different groups according to the raw materials employed to produce them and their biodegradability. The groups include conventional plastics, which are produced from monomers (chemical substances) and are not biodegradable, as well as plastics whose raw material comes from fossil fuels (petrobased plastics) and can be biodegraded. The other group comprises biobased plastics, which are synthesized from the biomass or from natural resources and are divided into two groups depending on whether they can be biodegraded or not. Except for conventional plastics, the other groups enter the bioplastics category [4,5].

One of the most widely used plastics in the farming sector is polyethylene (PE) [6,7,8,9]. Low-density polyethylene (LDPE) is a raw material that is generally employed to produce the plastic films utilized in most greenhouses and for the sacks used for storing special fertilizers [10]. In Europe, it has been estimated that 40,000 km^2^ of farmland is covered by these films [11]. LDPE is a synthetic hydrophobic high-molecular-weight polymer characterized by its hardness, resistance to chemicals, flexibility, and clearness [12]. These characteristics enable LDPE to be present in the industrial, farming, and domestic sectors [13]. As LDPE is a petrobased plastic that cannot be biodegraded in the short term, it remains in the natural environment for long periods of time [7,14]. Polyhydroxyalkanoates (PHAs) are a family of biobased plastics produced by different species of bacteria under nutrient-limiting (nitrogen and phosphorus) conditions with excess carbon [15,16]. Poly(hydroxybutyrate) (PHB) is the most studied polymer and is the best-known member of the PHAs family [17]. Poly(lactic) acid (PLA) has been proposed for producing horticultural products to reduce the environmental problems that are derived from the large quantities of the plastics employed in the sector [8,18,19]. It was the first polymer to be generated from renewable raw material that actually is widely commercialized on a large scale [15], and its properties are similar to those of polystyrene and polyethylene [20].

Today, conventional plastics are so widely employed in the agricultural sector because they possess the necessary mechanical properties to do so. However, in most applications, the retrieval and recycling of the employed plastics are impossible. So, plastic waste accumulates on farmland which, in Europe, corresponds to 5–6% of all the plastic waste on this continent [21]. This accumulation can affect the activity of different organisms, such as earthworms or microorganisms, and also physical soil properties (e.g., porosity and the structure of aggregates) [8,22]. They also act as a surface for different pollutants to be adsorbed, which means that these compounds might accumulate in soil [7,23]. Different studies considered using bioplastics as a solution to these sustainability problems [7,10,15,24,25,26,27,28,29,30,31,32,33] because they are produced from monomers (chemical substances) in a more energy-efficient way and can also be composted and recycled [25]. The main disadvantage of biobased plastics, as opposed to conventional plastics, is their low mechanical resistance [34]. It is for this reason that developing biodegradable plastics using an optimum combination between suitable mechanical properties and their own biodegradation represents a multidisciplinary challenge, as well as an opportunity to improve the sustainability of agricultural practices [24,27]. Carbon is one of the most important elements regarding soil productivity because different organisms (bacteria, fungi, and protozoa) use it as substrate to increase both their number and biomass [35]. Directly adding microorganisms (bioaugmentation) or organic amendments (biostimulation) is expected to facilitate biodegradation processes in soil [36,37]. Different studies investigated the role of amendments on the biodegradation of distinct polymers in soil [7,38], but have given inconsistent results. Therefore, more studies that clarify the role of not only biological activity, but also of the microbial communities that live in soil during plastics biodegradation processes, are necessary.

For all these reasons, the need to conduct studies to acquire information about plastics biodegradability and their final destination, and to develop strategies that help to remove plastics from the natural environment by reliable reproducible testing methods, is revealed. To do so, the specific objectives of the present work were as follows: (i) evaluate the biodegradability, in the short term, of three plastics widely used for various purposes, PHB, PLA, and LDPE, by following the procedure described in the ISO 17556 Standard [39], which includes the necessary procedures for measuring the biodegradation of plastics in soil; and (ii) study the effect of adding organic amendment to soil on the biodegradation of the selected plastics.

## 2. Materials and Methods

### 2.1. Soil

The soil sample employed to conduct this work corresponds to a Bt horizon of a calcic xeralf (Alfisol-type soil) situated on the mountainous Mondúver massif (La Safor, Valencia, Spain). Its location is as follows: 39°02′24.3″ N 0°21′14.5″ W. Soil was described in the field and was sampled following the procedure described by the FAO [40]. The field sample was duly registered in the laboratory. Then, it was spread over a plastic tray and plant materials and coarse fragments were separated. It was dried at 40–50 °C in a drying chamber and ground with a wooden rolling pin to be sieved at 2 mm. This allowed an air-dried fine earth fraction to be obtained to perform the analyses (FEF). Moisture content was determined by a gravimetric procedure. The pH was measured in 1:5 soil:water suspension (*v*/*v*) using a GLP pHmeter (Crison™, Barcelona, Spain) [41]. Electric conductivity (EC) was measured in an aqueous extract at 1:5 soil:water (*w*/*v*) by a PC 2700 conductimeter (EUTECH™ Instruments, Singapore) [42]. Particle size was determined by the Bouyoucos densimeter method [43]. Organic matter (OM) was determined by oxidation with potassium dichromate in the presence of sulphuric acid and by titration with ferrous ammonium sulphate [44]. Total nitrogen (N) was measured by an elemental EA 1110 CNHS Auto-Analyser (CE Instruments, Milan, Italy) with heat treatment at a temperature of at least 900 °C in the presence of oxygen following an ISO procedure [45].

### 2.2. Organic Amendment

The organic amendment employed in this experiment corresponds to a widespread organic amendment in agriculture: cow manure (CM). It was supplied by Desco S.L. company (Valencia, Spain). Moisture was determined by gravimetric procedure. pH and EC were determined following norms UNE-EN 13037 [46] and UNE-EN 13038 [47], respectively. OM was determined according to the procedure described in norm UNE-EN 13039 [48], while the Walkley and Black method [44] was followed for oxidizable organic carbon. Finally, to determine N content, the procedure described in ISO 13878 [45] was followed. The dose applied in the experiment was equivalent to 20 tons of manure per hectare within the range of manure application recommended in agriculture (between 10,000 and 40,000 kg ha^−1^) [49]. The contributed N dose corresponded to a quantity of 200 kg ha^−1^, which is slightly higher than recommended (170 kg ha^−1^).

### 2.3. Plastics and the Reference Material

The plastics used in this experiment were as follows: PHB (C_4_H_6_O_2_); PLA (C_3_H_4_O_2_); and LDPE (C_2_H_4_). The plastics were supplied by AIMPLAS—Plastics Technology Centre (València, Spain). The reference material (MR) was microcrystalline cellulose (C_6_H_10_O_5_), and these molecular formulas are constitutional repeating units. Oxidizable organic carbon was determined for each plastic, as well as the theoretical oxygen demand (ThOD), which is understood as the stoichiometric quantification of the oxygen required to completely oxidize a compound [50] that gives way to CO_2_ and H_2_O. In our case, ThOD is given by the stoichiometry of the following chemical reactions:

$$PHB:C_{4}H_{6}O_{2}+\frac{9}{2}O_{2}\to 4{CO}_{2}+3H_{2}O$$

$$PLA:C_{3}H_{4}O_{2}+3O_{2}\to 3{CO}_{2}+2H_{2}O$$

$$LDPE:C_{2}H_{4}+3O_{2}\to 2{CO}_{2}+2H_{2}O$$

$$Microcrystalline\;cellulose\left(MR\right):C_{6}H_{10}O_{5}+6O_{2}\to 6{CO}_{2}+5H_{2}O$$

### 2.4. Experimental Design

**Figure 1:**
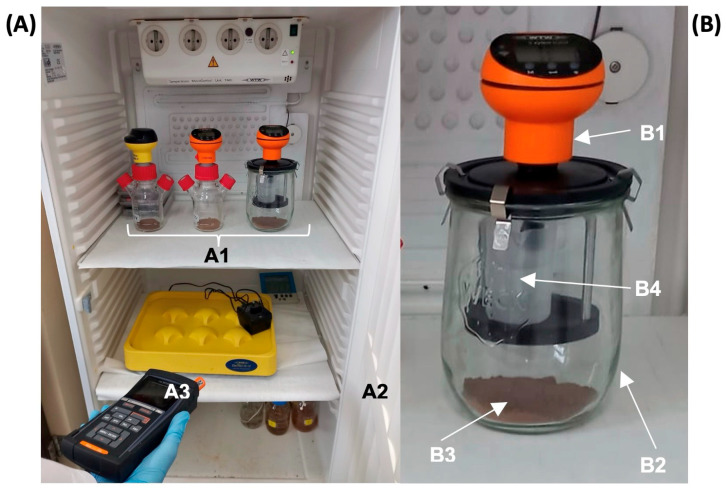
(**A**) Equipment used in the study: different OxiTop^®^ measuring systems used in the experiment (A1) inside the thermostatized chamber (A2), and the communication handheld for the data acquisition (A3). (**B**) Detail of the OxiTop^®^ device: respirometric measuring unit (B1), airtight glass vessel (B2), amended soil samples with plastics (B3), and baker with NaOH for CO_2_ trap (B4).

The procedure applied to carry out the following test is described in norm ISO 17556 [39], which includes the necessary procedures to measure the ultimate aerobic biodegradability of plastic materials in soil by measuring biochemical oxygen demand (BOD) in a respirometer or the amount of generated CO_2_. The norm can be applied to natural or synthetic polymers, copolymers, or mixtures of polymers. In our case, the applied method was to determine BOD with a respirometer. This study evaluated the biodegradation in soil of PHB, PLA, and LDPE with different treatments. The used reactors were respirometers (WTW^®^, OxiTop™-C, and OxiTop™-IDS, Weilheim, Germany) (Figure 1), which indirectly measure the quantity of consumed oxygen. The employed soil corresponded to FEF. Two blanks were made, one only with soil (Bl−) and the other with both soil and CM (Bl+), and a test was run with soil by adding CM as the MR and another in which soil, plastic, and CM had been biologically inactivated in an autoclave (Inact). For all the plastics, two treatments were carried out (Table 1): one with soil + plastic (−) and another with soil, plastic, and CM (+). For PHB: PHB− (soil with PHB) and PHB+ (soil with PHB and CM). For PLA: PLA𢈒 (soil with PLA) and PLA+ (soil with PLA and CM). For LDPE: LDPE𢈒 (soil with LDPE) and LDPE+ (soil with LDPE and CM). To each container, 21 mL of water was added, which corresponded to 60% of this soil’s water holding capacity (WHC), which was 35%. The exact quantities of soil, plastic, CM, and water added to each container are shown in Table 1.

The plastic material was introduced in pieces that were no bigger than 5 mm × 5 mm in size. For biological inactivation purposes, the corresponding containers were sterilized in an R2LPS autoclave (J.P. SELECTA^®^, Barcelona, Spain) at 121 °C for 20 min along with the parts required to place the respirometer, except for the head. Once autoclaved, the parts and the head were placed inside a laminar flow chamber superficially sterilized with UV light to start the experiment. Containers were left in the dark at 21 °C ± 1 °C in an incubator (WTW^®^, TS606/3, Weilheim, Germany) for 20 days.

### 2.5. Calculations

Biodegradability was determined according to the expression in Equation (1) [39]:

(1) $$D_{t}=\frac{{DBO}_{T}-{BOD}_{B}}{ThOD\times \rho_{T}}\times 100$$

where D_t_ is the percentage of the test material’s (plastic) biodegradation in time t; DBO_T_ is the BOD that corresponds to the container containing the plastic material that is expressed as milligrams of consumed oxygen per kilogram of dry soil (mg O_2_ kg^−1^); BOD_B_ is the BOD that corresponds to the control blank (mg O_2_ kg^−1^), which is the test soil; ThOD is the theoretical oxygen demand of the tested material and is expressed as milligrams of oxygen per gram of plastic material (mg O_2_ g^−1^); and ρ_T_ is the concentration of the test material (plastic) in the reaction mixture inside the container, expressed as g kg^−1^.

For the soil treatments with only soil (PHB−; PLA−; LDPE−; and the MR), Bl− was deducted, while Bl+ was deducted from the treatments with CM (PHB+; PLA+; and LDPE+). The difference was divided by the ThOD of the corresponding plastic by multiplying it by the quantity of this added plastic in the two replicas (Equation (1)).

The amount of consumed oxygen was obtained indirectly by measuring the negative pressure that took place inside the respirometer, which included a CO_2_ trap by adding NaOH lentils. Both consumed oxygen and the soda trap caused depression, which was captured by the head for short time intervals throughout the incubation time. After 20 experiment days, the results were obtained, which show decreased pressure in hectopascal units (hPa). The pressure values expressed as hPa were converted into atmospheres (atm), and temperature into Kelvin (°K) grades. Using the ideal gases equation (Equations (2)) and (3), the consumed O_2_ was calculated for each time in all the treatments and expressed per kg of dry soil.

(2) PV=nRT

(3) $$mgO_{2}=\frac{PV32}{0.082T}\times 1000$$

where P is pressure (atm); V is the container’s gas volume (L); n is the number of moles; R is the constant of ideal gases (L atm mol^−1^ °K^−1^); and T is temperature (°K).

Additionally, the change in the weight of the plastics between the beginning and the end of the experiment was determined. The different strips were washed and weighed before and after the process to determine the mass loss.

### 2.6. Statistical Analysis

All the calculations were conducted using Microsoft Office-Excel™ (version 2017). To determine differences in the results between treatments, a one-factor ANOVA was used with Tukey’s *post hoc* test. The employed statistical package was SPSS^®^ v28 (IBM^®^, Armonk, NY, USA).

## 3. Results and Discussion

### 3.1. Materials’ Characterization

#### 3.1.1. Soil Properties

According to the sampling, the profile presented the following genetic horizons: Ah, AB, Bt, and R. Soil were slightly basic (pH = 7.49). The moisture content ranged from 22.5% to 24.0% during the experiment. There were no salinity problems because the EC value was 0.127 dS m^−1^ (< 0.2 dS m^−1^). Volatile matter content was 2.36%. Soil organic matter content was 3.05%, and the soil’s texture class is clayey with 56% clay, 30% silt, and 14% sand; in this sense, Briassoulis and Mistriotis (2018) [28] indicate that fine texture natural soils are ideal for biodegradation tests of bio-based materials.

#### 3.1.2. Characteristics of Organic Amendment

The moisture content in the organic amendment employed in the tests was 60%. The percentage of OM, oxidizable organic carbon, and volatile matter of CM was 83%, 28.17%, and 48.73%, respectively.

#### 3.1.3. Plastics and the Reference Material

The plastic with the highest percentage of oxidizable organic carbon was PHB with 39.11%, followed by PLA with 13.25% and LDPE with 0.72%. As for the ThOD, the compound that required the most oxygen per gram was LDPE (3420 mg O_2_ g^−1^), followed by PHB (1670 mg O_2_ g^−1^), PLA (1330 mg O_2_ g^−1^), and microcrystalline cellulose (MR) (1185 mg O_2_ g^−1^).

### 3.2. O_2_ (BOD) Consumption

The amount of consumed O_2_ over the 20 days that the experiment lasted in each treatment shown is in Table 2. All the data are expressed as mg of consumed O_2_ per kg of dry soil (d.w.). As expected, in all cases in the sterilized material (Inact), consumed oxygen remained very low, and almost constant throughout the experiment (between 96 mg kg^−1^ at the beginning and 129 mg kg^−1^ at the end of the experiment). It is also important to stress that no sign of any alteration was noted in plastics. For the other treatments, the plastic-free control soil without amendment (Bl−) displayed the least biological activity, and consumed accumulated oxygen was 561 mg kg^−1^. The consumed oxygen in the soil with the CM amendment (Bl+) was 980 mg kg^−1^, which was almost double that of the control soil and was slightly higher than that in the MR soil, whose value was 823 mg kg^−1^. These results indicate that adding both CM and the MR stimulates soil’s biological activity.

The soil treatments without amendments with PLA (PLA−) and LDPE (LDPE−) showed intermediate and slightly greater activity than Bl−. There were no significant differences for the oxygen consumed between both treatments (Table 3), with 590 mg kg^−1^ and 646 mg kg^−1^, respectively, for PLA- and LDPE−. For the other treatments, PHB+, PHB−, PLA+, and LDPE+ led to a greater edaphic microbiota response, with consumed oxygen values of 1006 mg kg^−1^, 1000 mg kg^−1^, 936 mg kg^−1^, and 1012 mg kg^−1^, respectively. There were no significant differences among any of the treatments (Table 3). Although these values were not much higher than those of the soil with amendment (Bl+), they indicated that adding manure and/or plastics PHB and PLA also stimulated soil’s biological activity. It is also important to stress soil’s positive microbial response to the addition of PHB, as reflected in the oxygen consumption of treatment PHB- (1000 mg kg^−1^).

**Figure 2:**
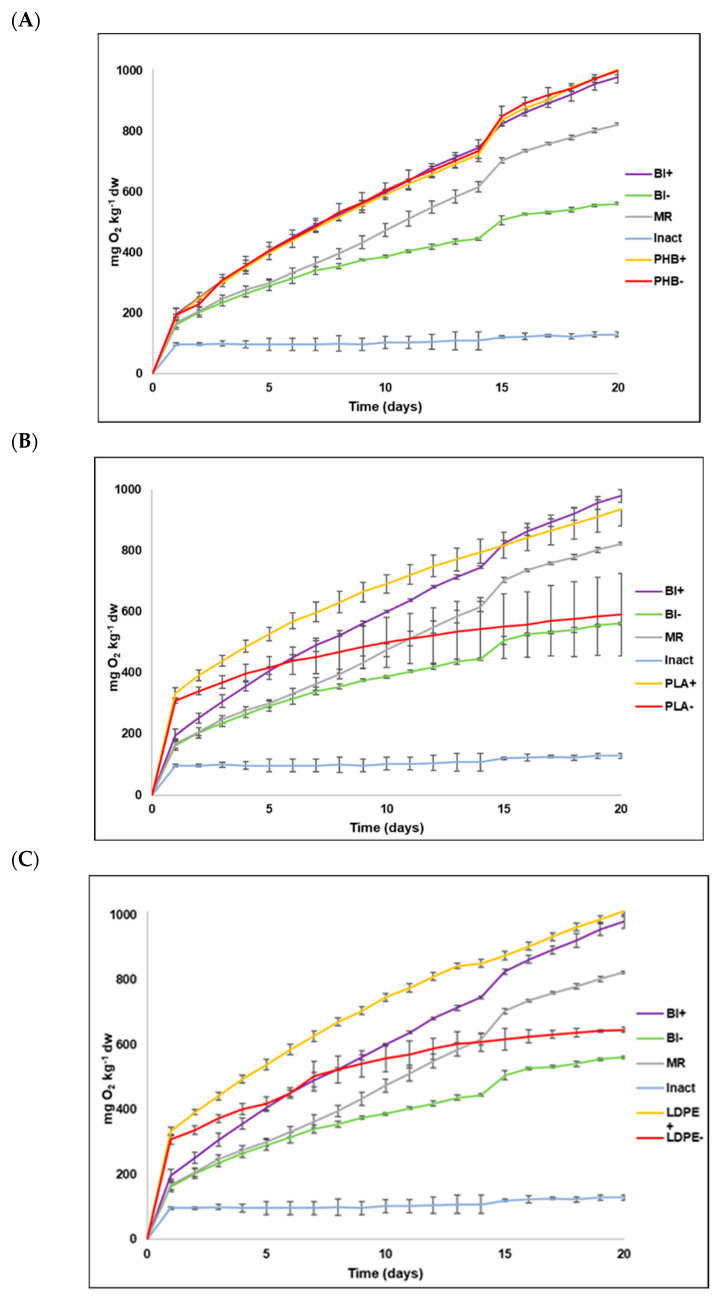
Evolution of oxygen consumption in soil with plastic materials and their different treatments during 20 days of incubation. (**A**) PHB; (**B**) PLA; and (**C**) LDPE. Bl, blank; MR, reference material; Inact, biologically inactivated; −, without organic amendment; +, with organic amendment added.

The comparison made of oxygen consumption evolution in soil to the different treatments and for the three plastic types is shown in Figure 2. For PHB (Figure 2A), no change whatsoever was noted for the sterile soil (Inact). The untreated soil (Bl−) presented the least activity, followed by the soil with the MR. The response of the other treatments (Bl+, PHB+ and PHB−) was greater and similar for each treatment. As previously indicated, soil biological activity responds in the same way to the additional adding of both CM (PHB+) and PHB (PHB−), and both jointly and separately. This finding revealed that this polymer derived from poly(hydroxybutyrate) acid very positively influenced soil biological activity stimulation. This was logical because the carbon content that was easily oxidizable in this plastic was higher than that of manure (see Section 3.1.3). With PLA (Figure 2B), for the first 15 days, the soil with both plastic and CM (PLA+) was the treatment with the most consumed oxygen, although in the end it was similar to the soil with amendment (Bl+) and the soil with microcrystalline cellulose (MR). The soil with plastic alone (PLA−) and the control soil (Bl−) consumed approximately half the oxygen that the previous treatments did, with the first treatment consuming more than the control soil. This may also be due to the oxidizable C content (13.25%) of this plastic material. Regarding LDPE (Figure 2C), as with PLA, the soil with this plastic and CM (LDPE+), and the soil with amendment (Bl+) followed by the MR, were the treatments that led to a greater microorganisms’ response. For the first 15 days, although the consumption in LDPE+ was slightly higher, these three treatments obtained similar values. Similarly to what occurred with PHB and PLA, the soil with the MR obtained a slightly lower value than that for LDPE+ and Bl+, which confirms the effect of organic amendment on soil’s biological activity. Except for the sterile soil (Inact), and as in previous cases, the poorest response was obtained for the soil with only plastic (LDPE−) and the control soil (Bl−).

The fact that the Inact treatment presented slightly higher consumed oxygen in the first days, which then remained practically invariable for the rest of the time (Table 2 and Figure 2), firstly suggests that the autoclaving process was correctly performed, and secondly, that the small amount of consumed oxygen in these containers was not caused by biological activity, but by some short-lasting physicochemical mechanism. When comparing the amount of consumed oxygen between this treatment (129 mg kg^−1^) and the control soil (Bl−, 561 mg kg^−1^), physicochemical degradation played an imperceptible role, unlike the biological degradation that was the main process to eliminate these polymers in soils, as previously demonstrated in different studies [4,13,16,21,22,51,52].

Another point to highlight is the fact that except for PLA+, the consumed oxygen in all the treatments was above that of its corresponding blank (Table 2). For the soil with amendment (Bl+), the quantity of consumed oxygen almost doubled that of the soil with no amendment (Bl−) because organic amendment was added. Indeed, adding CM very possibly led to bioaugmentation and/or biostimulation processes that, on the one hand, acted as an input of microorganisms and, on the other hand, acted as a source of nutrients that stimulated microbial activity. Such an effect has been described by different studies: for instance, Hamarashid et al. (2010) [35] indicate that organic C is fundamental for bacteria, fungi, and protozoa in soil, which use it as substrate to increment both their number and biomass. Chaturvedi et al. (2013) and Li et al. (2013) [36,37] observed that directly adding microorganisms or organic amendments facilitates biodegradation processes in soil.

Our results also indicate that adding cellulose (MR) to soil led to the slightly lesser stimulation of edaphic microbiota than that achieved by adding PHB (Figure 2), which confirmed the results of other previous studies [16,21], and suggests that PHB is a highly biodegradable polymer. Indeed, it has been used as a positive control in many tests and plays the same role as microcrystalline cellulose did in the present study [21]. The similar oxygen consumption in both treatments (1006 mg kg^−1^ for PHB+ and 1000 mg kg^−1^ for PHB−) also denotes high compound biodegradability because the soil with this plastic and without amendment (PHB−) consumed practically the same quantity of oxygen as the treatment with CM (PHB+). This situation did not occur in PLA and LDPE. In these cases, the treatments with CM (PLA+ and LDPE+) consumed almost double the oxygen than the soils with no amendment (PLA− and LDPE−). As previously mentioned, the fact that oxygen consumption increased when CM was added is related to bioaugmentation and biostimulation processes because adding manure should increase both the microbial population and nutrient content, especially C and N, which considerably stimulates microbial activity.

### 3.3. Plastic Material Biodegradability

**Figure 3:**
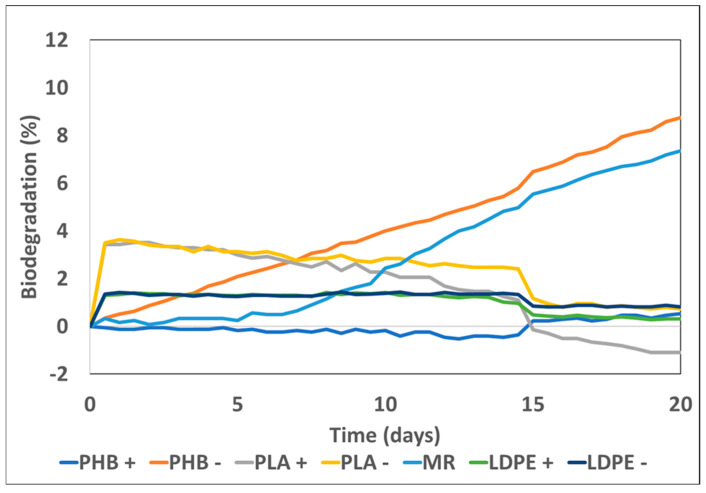
Evolution of plastic biodegradation in soil during the 20-day test. PHB+: soil with PHB and cow manure (CM); PHB−: soil with PHB only. PLA+: soil with PLA and CM; PLA−: soil with PLA only. MR: soil with reference material (microcrystalline cellulose). LDPE+: soil with LDPE and CM; LDPE−: soil with LDPE only.

Table 3 shows the plastics biodegradation percentages obtained at the end of the experiment. For the MR, which acted as a positive control, biodegradation was 7.37%. Significant differences were found for PHB between the treatment with CM (PHB+) and without CM (PHB−), whose biodegradation percentages were, respectively, 0.52% and 8.76%. The PHB+ biodegradation percentages were slightly higher than those of cellulose (MR), respectively, with 8.76% and 7.37%, which confirmed high PHB biodegradability. These results are similar to those reported by other authors [16,21,22,43,53,54,55,56,57]. With PLA, the biodegradability in the soil with (PLA+) and without (PLA−) amendment was −1.10% and 0.73%, respectively. The fact that negative values were obtained is explained by the bigger quantity of oxygen consumed in the control soil; that is, the control value is subtracted from the problem sample value to eliminate the part of the oxygen consumed by soil; this result has also been indicated by Briassoulis and Dejean (2010) [58] in their experiment to measure biodegradation in soil with different plastic films. Those authors also noted greater CO_2_ production in blanks than in soils with plastics. This biodegradability evolution shown in Figure 3 for PLA, along with such low biodegradability percentages, is interesting for being a polymer that is classified as biodegradable. In line with this, both Rudnik and Briassoulis (2011) and Tokiwa and Calabia (2006) [15,20] indicate that the biodegradation of this polymer needs higher temperatures, such as those required to accomplish composting processes (60 °C).

Slezak et al. (2023) and Wang et al. (2023) [9,19] also indicated that this plastic presents low biodegradability at ambient temperature. Many studies evaluated PLA biodegradability in soils under similar conditions to those herein described with similar results. Kamiya et al. (2007) [59] did not observe PLA degradation in soil for 120 days, and neither did Hoshino et al. (2001) [60], who did not detect PLA degradation in soil for 90 days at any of the 19 Japanese locations where tests were performed. Adhikari et al. (2016) [61] measured PLA biodegradability under similar conditions to ours (in soil for 28 days at 25 °C) and reported a biodegradation percentage of only 13.8% at the end of their experiment. Harmean et al. (2014) [62] reported a biodegradation percentage of 37.4% after 56 days at 30 °C with 80% humidity. Nevertheless, those studies that evaluated PLA biodegradability in compost at higher temperatures obtained a much higher biodegradation percentage. Kale et al. (2007) [63] also evaluated biodegradability in compost for 58 days at 58 °C and obtained a biodegradation percentage of 84%. Likewise, Mihai et al. (2014) [64] reported 60% PLA biodegradation in compost after exposing it to 58 °C for 30 days. As soil temperatures do not exceed 30 °C under normal conditions and increasing such soil temperatures is unfeasible, PLA biodegradation in soils would very much depend only on time [9,15] and, to a certain extent, on soil characteristics. To determine if this polymer is a good candidate to safely and sustainably replace the conventional plastics employed in agriculture, more research is needed to accurately find out the time needed to complete PLA biodegradation under different conditions.

For the treatments with LDPE (LDPE+ and LDPE−), and as Figure 2 depicts, the biodegradation percentages remained constant at very low levels for 20 days. At the end of the experiment, the biodegradation percentages (Table 3) for both these treatments were 0.31% for LDPE+ and 0.82% for LDPE−, which confirms the low biodegradability of this plastic, a characteristic that has been demonstrated before by different authors [7,8,13,21,65,66].

BOD_T_, biological oxygen demand (BOD) corresponding to the plastic material; ρT, concentration of the plastic material in the mixture; ThOD, theoretical oxygen demand of plastic; and D_t_, percentage of biodegradation of the plastic material in time t = 20 d. BOD_T_ and D_t_ values with the same letter indicate that there are no significant differences between treatments (n = 3, Tukey *p* < 0.05). PHB+. soil plus PHB and organic matter; PHB−. soil plus PHB; PLA+. soil plus PLA and organic matter; PLA−, soil with PLA; LDPE+, soil with LDPE and EV; LDPE−, soil with LDPE; and MR, soil with reference material (microcrystalline cellulose).

Its biodegradability is still very low, even when adding microbial inoculum [66]. Those authors concluded that oxidation prior to LDPE biodegradation is the only option for its alteration, which leads to not only the formation of more attackable monomers, but also to the application of the microbiome from rubbish dumps. In accord with these results, Beltrán-Sanahuja et al. (2021) [6] conclude that after one year of exposure to soil with different environmental conditions, conventional materials showed no degradation through several lines of evidence: weight loss, spectroscopy, and thermal derived metrics.

### 3.4. Effect of Organic Amendment on Plastics Biodegradation

Regarding the effect of adding organic amendments to the biodegradation of plastic materials in soils, it has been shown that soils rich in OM present greater microbial activity [60,67], and the amount of OM in soil has been positively correlated with material biodegradation [7,35,38]. So, a soil with CM amendment is expected to present a higher biodegradation percentage. Furthermore, the previous section indicates its positive effect on increasing oxygen consumption because biological activity increases. However, it was surprising to note that the soils with plastics and CM amendment herein employed (PHB+, PLA+, and LDPE+) obtained much lower biodegradation percentages than their counterparts with no amendment (PHB−, PLA−, and LDPE−) (Table 3). Thus, the oxygen consumption data are not reliable enough for us to discuss the possible benefit of organic amendments during plastics biodegradation processes. The low plastics biodegradability in the reactors with CM might be due to the soil microorganisms that come from a natural environment not having acclimatized in the short term and preferring to consume the source of C from CM that is more easily oxidizable, and thus, plastics would not be attacked. In fact, the main difference found to the other works consulted in this study was that the inocula of the used microorganisms came from isolates from rubbish dumps [66,68,69], which have undergone a long acclimatization process, whereas microorganisms came from a natural environment in our case. Roy et al. (2008) [70] also mentioned in most studies into the plastics biodegradation that specific and very well-identified bacterial strains have been employed. Yet, under natural conditions, neither the composition nor the efficiency of microbial communities can be anticipated, nor can their catalytic abilities [7,51]. Sen and Raut (2015) [13] indicated that one of the strategies used to facilitate the dissolution and consequent degradation by microorganisms is to use the polymer as the only source of C. Indeed, Park and Kim (2019) [71] observed 14.7% weight loss in PE particles when they introduced it as the only source of C during a 60-day experiment.

In the present work, the PHB biodegradation percentage in the treatment with CM was 0.52%, while it was 8.76% in the soil with no amendment (Table 3). There are also other factors that may have an influence and could also be due to the microbial activity that derives from adding OM not implying greater polymer biodegradation. Microorganism activity may increase when OM is added to the medium because microorganisms employ this same OM as a source of C, and not the biodegradable polymers present. Hoshino et al. (2001) [60] demonstrated that the biodegradation of polymers in soil is affected mainly by temperature and the total available N content in soil, whereas adding organic supplements does not offer any benefit. Given these differences in the results and the few studies found about the effect of organic amendments on plastics biodegradation processes, further research is necessary to explain the complex relations that occur among microbial activity, OM, and the biodegradation of polymers in soil.

**Figure 4:**
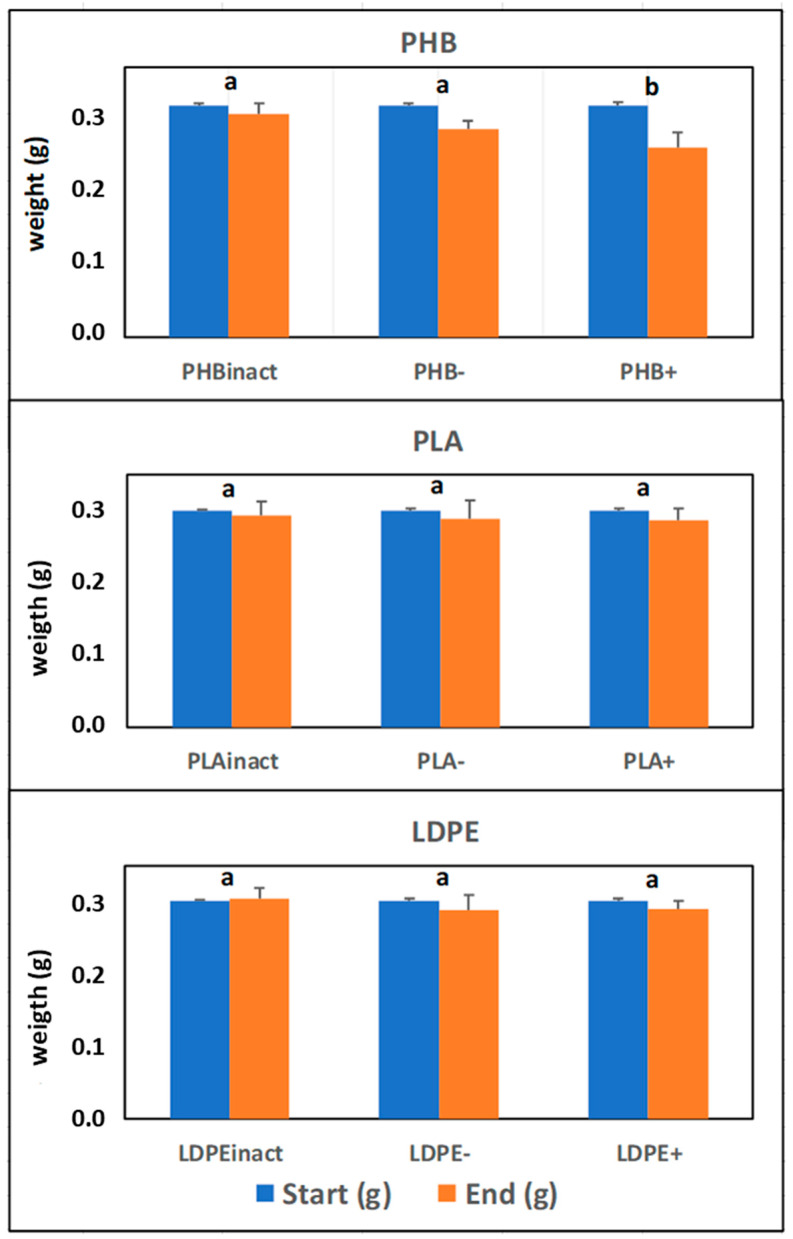
Mass of plastic materials at the start and end of the experiment. Biologically inactivated (Inact); with organic matter added (+), without organic matter (−). Different letters indicate significant differences at α < 0.05 after *post hoc* Tukey test.

**Figure 5:**
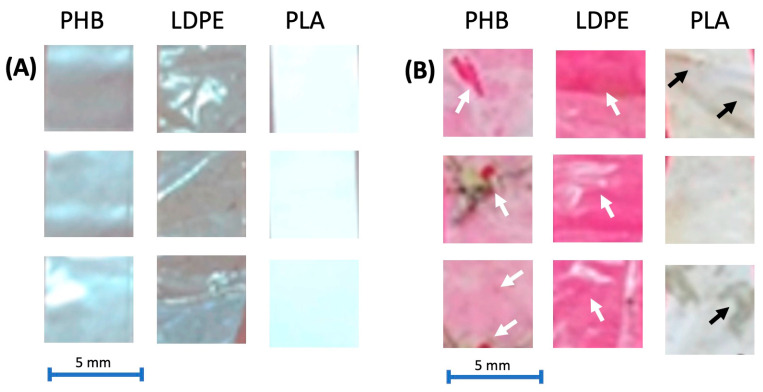
Aspects of three different plastics (PHB, LDPE, and PLA) used in the study along the exposure time (**A**) at the beginning of the experiment (0 days) and (**B**) at the end of the experiment (20 days). Black and white arrows indicate holes in the plastic slides due to degradation. A pink background has been used in order to improve the contrast.

To complete this study, Figure 4 shows the weight values of the plastics in each treatment at the beginning and end of the experiment. As observed, an important decrease in plastic weight variation was only obtained in the case of PHB: 10.0% for PHB−, and particularly significant in the treatment with manure addition (18.4%). No significant differences were observed for PLA and LDPE. This finding confirms the previous results and highlights the different biodegradability of these plastic materials. Figure 5 highlights stark differences in the degradation of the three plastics between the start and end of the experiment: PHB exhibited small holes, indicative of microbial/enzymatic attack or hydrolysis, and consistent with its known biodegradability; this aligns with observed O_2_ consumption, biodegradation percentages, and weight loss, confirming expected breakdown mechanisms, while LDPE and PLA remained virtually intact, showing minimal degradation. Their persistence correlates with low O_2_ uptake and negligible weight loss, underscoring their environmental recalcitrance. The results emphasize PHB’s potential as an eco-friendly alternative, while LDPE and PLAs are virtually intact.

## 4. Conclusions

After evaluating the biodegradability of the three conventional plastic materials (PHB, PLA, and LDPE) in a Mediterranean Alfisol-type soil and the effect of adding organic amendment (CM) by the method based on norm ISO 17556 [39], which is the international norm for measuring the biodegradation of plastics in soil; our results confirm that measuring only O_2_ consumption from soil biological activity is not sufficient to evaluate biodegradation effects. PHB behaves similarly to the positive MR (microcrystalline cellulose) compared to the PLA and LDPE that barely degrade. Our findings confirm too that the biodegradability order of these three plastic materials is PHB >>> PLA > LDPE.

PHB could be a good alternative for replacing conventional farming plastics owing to its high biodegradability in soil in the short term. Conversely, PLA is not a good candidate for replacing conventional plastic materials in the agricultural sector because its biodegradation takes place at temperatures that cannot be naturally reached on farmland. LDPE is a polymer whose biodegradability is very low, and therefore, must be ruled out for any use. Adding CM to our soil did not influence the biodegradability of these three plastic materials. This finding suggests that natural soil biodiversity seems only high enough to permit materials’ biodegradation without having to add amendments of this type. More studies are needed to elucidate how and in what way organic amendments should be applied to facilitate biodegradation processes and to understand the complex relations among microbial communities, soil, and polymer biodegradation mechanisms.

Finally, this study indicates that in order to solve today’s plastics problems, it is necessary to accurately know the biodegradation conditions of the plastics chosen as an alternative so that use does not harm the natural environment, which is currently the case of conventional plastics. There is also the need to more widely investigate the short- and long-term destinations of bioplastics within soil environments because both the nature and properties of soils can significantly impact their biodegradation. Therefore, more studies are necessary to understand these variables in detail. Future research should incorporate enzyme activity assays and metagenomics to gain deeper insights into biodegradation mechanisms. While biodegradable plastics offer a potential alternative to conventional plastics, they alone cannot fully resolve the contamination crisis. A holistic approach—emphasizing prevention, reduction, and in situ reuse—must be prioritized to minimize plastic pollution at its source.

## Figures and Tables

**Table 1:** Treatments and amount of soil, organic matter (OM), type of plastics, and water introduced into each vessel.

Treatment	Replicate	Soil (g)	OM (g)	Plastics (g)	Water (mL)
Bl−	1	100	1,43	0.0000	21
	2	100	1.43	0.0000	21
Bl+	1	100	0.00	0.0000	21
	2	100	0.00	0.0000	21
MR	1	100	0.00	0.3005	21
	2	100	0.00	0.3005	21
Inact	1	100	1.43	0.3001	21
	2	100	1.43	0.3004	21
PHB+	1	100	1.43	0.3000	21
	2	100	1.43	0.3002	21
PHB−	1	100	0.00	0.3007	21
	2	100	0.00	0.3007	21
PLA−	1	100	1.43	0.3006	21
	2	100	1.43	0.3004	21
PLA−	1	100	0.00	0.3009	21
	2	100	0.00	0.3003	21
LDPE+	1	100	1.43	0.3008	21
	2	100	1.43	0.3004	21
LDPE−	1	100	0.00	0.3004	21
	2	100	0.00	0.3005	21

**Table 2:** Amount of O_2_ consumed in each treatment with the three plastics over the 20-day trial (mg O_2_ kg^−1^ dw) (standard deviation).

Treatment	24 h	End (20 Days)
Inact	96 (4)	129 (8)
Bl−	163 (16)	564 (4)
Bl+	196 (21)	980 (24)
MR	169 (16)	823 (8)
PHB+	190 (6)	1006 (8)
PHB−	195 (21)	1000 (13)
PLA+	336 (13)	936 (54)
PLA−	201 (10)	590 (36)
LDPE+	333 (13)	1012 (17)
LDPE−	178 (15)	646 (8)

Inact, sterilized material; Bl−, plastic-free control soil without amendment; Bl+, plastic-free control soil with amendment (CM); MR, soil with reference material; PHB−, PLA−, LDPE, and soil treatments with plastics without amendments; and PHB+, PLA+, LDPE+, and soil treatments with plastics and amendment.

**Table 3:** Biodegradation of PHB, PLA, and LDPE with (+) and without (−) organic amendment after 20 days of incubation in a Mediterranean xeralf soil. Same letter indicates nonsignificant differences in the *post hoc* ANOVA test.

Treatment	BODT	BODB	ThOD	ρT	Dt
PHB+	1006 ^a^	980	1670	3.00	0.52 ^b^
PHB−	1000 ^a^	561	1670	3.00	8.76 ^a^
PLA+	936 ^a^	980	1330	3.00	−1.10 ^b^
PLA−	590 ^c^	561	1330	3.00	0.73 ^b^
LDPE+	1012 ^a^	980	3420	3.00	0.31 ^b^
LDPE−	646 ^c^	561	3420	3.00	0.82 ^b^
MR	823 ^b^	561	1185	3.00	7.37 ^a^

## Data Availability

The datasets used and/or analyzed during the current study are available from the corresponding author upon reasonable request.
